# Autism Spectrum Disorder and Attention-Deficit/Hyperactivity Disorder: Shared or Unique Neurocognitive Profiles?

**DOI:** 10.1007/s10802-022-00958-6

**Published:** 2022-08-25

**Authors:** Russell J. Schachar, Annie Dupuis, Paul D. Arnold, Evdokia Anagnostou, Elizabeth Kelley, Stelios Georgiades, Robert Nicolson, Parker Townes, Christie L. Burton, Jennifer Crosbie

**Affiliations:** 1 Department of Psychiatry, The Hospital for Sick Children, Neurosciences and Mental Health, 555 University Avenue, Toronto, ON M5G 1X8 Canada; 2 Department of Psychiatry, Faculty of Medicine, University of Toronto, 27 King’s College Cir, Toronto, ON M5S 1A1 Canada; 3 Department of Biostatistics, Dalla Lana School of Public Health, University of Toronto, 27 King’s College Cir, Toronto, ON M5S 1A1 Canada; 4 The Mathison Centre for Mental Health Research & Education, 3280 Hospital Dr NW, Calgary, AB T2N 4Z6 Canada; 5 Holland Bloorview Research Institute, 150 Kilgour Road, Toronto, ON M4G 1R8 Canada; 6 Departments of Psychology and Psychiatry, Queens University, 99 University Ave, Kingston, ON K7L 3N6 Canada; 7 Department of Psychiatry and Behavioural Neurosciences, McMaster University, 1280 Main St W, Hamilton, ON L8S 4L8 Canada; 8 Department of Psychiatry, Western University, 1151 Richmond St, London, ON N6A 3K7 Canada; 9 Department of Paediatrics, Faculty of Medicine, University of Toronto, 27 King’s College Cir, Toronto, ON M5S 1A1 Canada; 10 Research Institute, The Hospital for Sick Children, 555 University Avenue, Toronto, ON M5G 1X8 Canada

**Keywords:** ADHD, ASD, Neuro-cognition, Inhibitory control, Reaction time variability, Stop-signal task

## Abstract

Attention-deficit/hyperactivity (ADHD) and autism spectrum (ASD) disorders are commonly co-occurring conditions characterized by neurocognitive impairments. Few studies have directly compared neurocognitive profiles in ADHD and ASD and fewer still have controlled for comorbidity of ADHD and ASD. All direct comparisons have been in clinic samples, leaving the question of generalizability of results unaddressed. We compared neurocognitive performance in clinically ascertained ASD (n = 261) and ADHD (n = 423) cases and controls (n = 162), 6.0–17.9 years of age. We also compared ASD (n = 190) and ADHD (n = 926) cases ascertained in the community with controls (n = 14,842) of similar age. Using the stop-signal task (SST), we measured response inhibition (stop-signal reaction time–SSRT), sustained attention (defined as reaction time variability–RTV), and reaction time (RT). We controlled for comorbidity using ADHD and ASD trait scores and categorically-defined ADHD. Compared with controls, both clinic ADHD and ASD had significantly longer SSRT and RTV than controls and did not differ from each other. ADHD traits accounted for neurocognitive impairment in ASD, but not vice versa. There were no group differences for RT. Similar patterns of neurocognitive impairment were observed in the community sample. In the largest direct comparison of ADHD and ASD to date, we found impaired response inhibition and sustained attention in both disorders. However, neurocognitive impairment in ASD was almost completely accounted for by comorbid ADHD. Results generalized in the community sample indicating that referral bias alone did not drive results. Response inhibition and sustained attention likely play a role in ADHD and ASD.

**Supplementary Information:**

The online version contains supplementary material available at 10.1007/s10802-022-00958-6.

## Introduction

Attention-deficit/hyperactivity disorder (ADHD) and autism spectrum disorder (ASD) are impairing, persistent and heritable neurodevelopmental disorders (APA, 2013) that share some genetic (Ghirardi et al., 2019; Lee et al., 2019; Polderman et al., 2014) and neural features (Boedhoe et al., 2020). However, it is not clear whether these disorders share neurocognitive profiles (Antshel & Russo, 2019). Delineating neurocognitive profiles for ADHD and ASD could aid diagnosis, clarify whether ADHD found in ASD is a phenocopy of ADHD and advance understanding of disorder mechanisms (Chang et al., 2020; Glahn et al., 2014). To date, most conclusions about the neurocognitive profiles of ADHD and ASD have been derived from indirect comparisons of each disorder with typically developing controls (e.g., ADHD versus controls–Lipszyc & Schachar, 2010; Pievsky & McGrath, 2018; Pineda-Alhucema et al., 2018; Wright et al., 2014 and ASD versus controls–Demetriou et al., 2018; Kaur & Pany, 2020). Results from these indirect comparisons suggest that ADHD, but not ASD, is characterized by poor response inhibition (Antshel & Russo, 2019; Corbett et al., 2009; Craig et al., 2015; Sinzig et al., 2009) and poorer sustained attention (reflected in greater reaction time variability–RTV), whereas those with ASD have worse cognitive flexibility and planning (Craig et al., 2015; Happé et al., 2006; Hosenbocus & Chahal, 2012; Lukito et al., 2020; Salcedo-Marin et al., 2013).

More compelling evidence for the uniqueness of the neurocognitive profiles of ASD and ADHD would come from direct comparisons that would allow control over variations in task conditions, performance metrics, testing environments (e.g., in a neuroimaging scanner) and demographic variables including age, sex, and medication status during testing (Buti et al., 2011). Importantly, direct comparison allows for systematic control over comorbidity, which is common between ASD and ADHD. ADHD is the most common comorbidity of ASD with comorbidity estimates of 40–70% (Brookman-Frazee et al., 2018; Joshi et al., 2017; Lyall et al., 2017). Subthreshold ADHD traits are also common in ASD (Pehlivanidis et al., 2020). While ASD traits are evident in ADHD, they are less common than ADHD traits in ASD (Nijmeijer et al., 2009; Rommelse et al., 2010). Given the well-established literature on neurocognitive impairment in ADHD such as in response inhibition and sustained attention (Karalunas et al., 2014; Pievsky & McGrath, 2018), failure to control for comorbid ADHD traits in neurocognitive studies of ASD could yield misleading conclusions.

A scoping review (in progress) identified 60 direct comparisons of neurocognition (Mar et al., in prep) in ADHD and ASD. There was considerable variation in the measures used, the indices of performance derived from these measures and the neurocognitive domains covered in these studies. Only five studies reported performance on the stop-signal task (SST), a widely used measure of response inhibition (Albajara Sáenz et al., 2020; Karalunas et al., 2018; Kuijper et al., 2015, 2017; Van Hulst et al., 2018). The largest of these studies (Karalunas et al., 2018) reported that ASD and ADHD had comparable deficits in response inhibition reflected in stop-signal reaction time (SSRT; also Van Hulst et al., 2018). Two studies found no group differences (Kuijper et al., 2015, 2017) and one study reported that ADHD, but not ASD, showed a deficit in response inhibition (Albajara Sáenz et al., 2020). Reaction time (RT) and RTV measured in the SST were longer in ASD than in ADHD, which were both longer than in controls in two of the three studies that reported these performance indices (Karalunas et al., 2018; Van Hulst et al., 2018 compare with Albajara Sáenz et al., 2020). Only the Karalunas et al. (2018) study controlled for comorbid traits and reported that longer SSRT, slower RT and greater RTV in ASD was not a function of comorbid ADHD. They did not find any effect of comorbid ASD traits on neurocognitive impairment in ADHD (Karalunas et al., 2018).

All direct comparisons of ADHD and ASD using the SST were conducted in clinic samples and each study had its own selection biases which, in some studies, differed for ADHD and ASD recruitment (cf. Karalunas et al., 2018) leaving unanswered the question of whether findings can be generalized beyond specific clinic settings (Low et al., 2008). Neurocognitive impairment could vary as a function of disorder severity or comorbidity in cases referred to specialty clinics (Pearce & Richiardi, 2014; Snoep et al., 2014). Clearly, the differences in SSRT, RTV and RT between ADHD and ASD require further direct comparison in both clinical and non-clinical samples.

We report the results of a direct comparison of SSRT, RTV and RT in rigorously assessed ADHD and ASD participants measured using the SST. We controlled for comorbid ADHD and ASD using validated trait measures and by comparison of ASD groups with and without comorbid ADHD. To test the generalizability of the results of the clinic sample, we also studied a community sample of individuals who reported a diagnosis of ADHD or ASD, where comorbidity was also controlled for.

## Method

### Clinic Participants

Clinic ADHD and ASD participants (age 6.0–17.9 years) were recruited from five different hospital- or university-based child and adolescent clinics. For ASD participants, recruitment into the study followed assessment and for ADHD participants, recruitment was concurrent with the initial assessment. Most participants received treatment in the community prior to and following research participation. Treatment involved a mix of medication, psychosocial intervention and school consultation. Each participant was assessed by a psychiatrist and/or a psychologist to confirm their primary diagnosis using the Autism Diagnostic Observation Schedule–2 (ADOS), Autism Diagnostic Interview–Revised (ADI-R) for ASD (Lord et al., 1994) and/or the Parent Interview for Child Symptoms (PICS) for ADHD (Ickowicz et al., 2006). Academic history and report cards when available were reviewed with informants as were medical, developmental and family history. Teachers and parents provided ratings of ADHD using the SWAN questionnaire. Clinicians reached consensus diagnosis using all available information. Anyone with an ASD diagnosis with or without comorbidity was classified as ASD (261). There were 423 participants with a diagnosis of ADHD (DSM-IV: APA, 1994 or DSM-5: APA, 2013). One-hundred and sixty-two controls were recruited by advertising in local hospitals and were assessed using the PICS to ensure that they did not have a mental illness. As described below, comorbidity was assessed using parent rating scales.

### Community Participants

‘Community’ participants were visitors (n = 16,720, aged 6.0–17.9 years) to a public science museum (Crosbie et al., 2013). Parents (n = 13,691) provided information about their child’s diagnosis, treatment, and behavior. When parents were unavailable, we used self-reports for those older than 12 years of age (n = 2,909). We excluded self-respondents younger than 12 years of age (n = 120), those who reported a community diagnosis of schizophrenia (n = 16) or obsessive–compulsive disorder (n = 280), and those reporting neuroleptic use but no diagnosis (n = 9). We could not collect ratings from teachers because of the setting and de-identification of participants. We classified participants as ASD (n = 190), ADHD (n = 926) or Controls (n = 14,842) based on a report of a diagnosis or treatment by a health care practitioner. To assess the role of ADHD trait severity independent of diagnosis, we identified a group of Community participants who did not report a diagnosis of ASD or ADHD but who had high ADHD trait scores (total, inattentive, and/or hyperactive traits above a t-score of 70, 98%ile for age on the SWAN scale) referred to as the Community ADHD High Trait group (n = 337). If participants did not meet criteria for the Community ADHD or ASD groups and did not have total, inattentive, and/or hyperactive traits above a t-score of 70 (corresponding to 98%ile for age on the Strengths and Weaknesses of Attention-Deficit/Hyperactivity Disorder Symptoms and Normal Behavior Scale–SWAN scale as described below, n = 337), they were included in the Community Control group.

### ADHD and ASD Trait and Symptom Measures

Parents rated their children on 18 ADHD items using the SWAN questionnaire (Burton et al., 2019; Swanson et al., 2012). They were instructed to rate behavioral traits over the previous 6 months from -3 far below average to + 3 far above average. From the SWAN scores, we reversed the scores and then calculated an age standardized t-score such that higher SWAN t-scores represented greater severity of ADHD traits (see Supplemental methods #1 for further details). We summed the number of ADHD symptoms rated as 2 or 3 and categorized ASD participants as having comorbid ADHD if they had 6 inattentive and/or 6 hyperactive-impulsive symptoms. Parent- and self-report SWAN scores showed high convergent validity with Conners' scales (rho = 0.72 for parent respondents and 0.71 for self-respondents) and high sensitivity and specificity (parent AUC = 0.85–0.88, self AUC = 0.71–0.75) for a community diagnosis of ADHD (Burton et al., 2019). Cut points established in the community sample discriminated ADHD clinic cases from controls with a sensitivity of 84% and specificity of 92%. High SWAN scores and scores above these cut points were significantly associated with polygenic risk for ADHD. SWAN scores were not associated with polygenic risk for OCD or anxiety disorders (Burton et al., 2019).

In the Clinic sample only, we used the Social Communications Questionnaire (Lifetime SCQ; Berument et al., 1999) to assess ASD symptoms. The SCQ consists of 39 items to which parents answered yes or no with higher scores representing more social communication impairment. The SCQ has good internal consistency (alpha = 0.90) and high validity for discriminating between ASD and non-ASD (AUC = 0.86).

### IQ and Medication Use

IQ measurement was available for Clinic participants only. Full-scale IQ was estimated using the age-appropriate Wechsler or Stanford-Binet scales. Informants in both the Community and Clinic samples reported medication usage within the previous 24 h prior to performing the SST. Recent use of medications was reported (see Supplemental Fig. 1 and Supplemental Table 1) and was categorized as stimulant (e.g., methylphenidate; dextroamphetamine) or non-stimulant medications (e.g., atomoxetine) used for ADHD, antipsychotic (e.g., risperidone), and specific serotonin reuptake inhibitors (SSRIs) which are used to treat anxiety, depression, and OCD. The proportion of ASD, ADHD and High Trait participants in the community and clinic samples who were using medication was virtually identical with the exception of higher ADHD medication use in the ASD clinic sample than in the ASD community sample (31.4 versus 19.5). None of the participants in the control samples were taking any medications (ADHD medications in community was part of the definition of community ADHD diagnosis). We controlled for stimulant medication in the models (below), but not for SSRI or neuroleptics given how infrequently they were reported in the absence of stimulants.

### Neurocognitive Measure

The stop signal task is a widely used measure of response inhibition (SSRT; see Supplemental methods #2 for task description). Participants were presented with either an X or an O (go stimuli) and instructed to respond as quickly and as accurately as they could using either their left (X) or right (O) hand (go trials). On 25% of random trials, a tone is presented via headphones following presentation of the go stimulus (stop signal). Participants were instructed to stop their response if they heard the stop signal. The task consisted of a practice block (24 trials; 18 go trials; six stop trials) and four experimental blocks of 24 trials for a total of 72 go trials and 24 stop trials. We calculated SSRT using interpolation (Verbruggen et al., 2019) and standard deviation of response times (RTV) across all go trials following a correct go response. RT on go responses following a correct go response estimates preparedness, perception and action (Rommelse et al., 2020). We considered the distribution of RT or RTV after correct-go responses only and not after all trial types because of skewing due to slowed responses after stop-inhibit (successful stopping) and stop-respond (unsuccessful stopping) trials (Dupuis et al., 2018). Those longer reaction times would confound and increase the mean RT and inflate RTV by adding between trial type differences to the estimate of overall variability. Supplemental Table 2 shows the proportion of participants in each group whose data were excluded because they failed the SST validity screen. More ADHD, ASD and ADHD High Trait participants were excluded than Control participants in Community and Clinic samples.

### Statistical Analyses

Statistical analyses were performed using SAS 9.4 (2012). Linear regression models used a log transformation of the SST outcomes to correct heteroscedasticity across age (higher variability in conjunction with higher scores at younger ages – e.g., Community SSRT SD at age 6 was 198 ms compared to Community SSRT SD at age 17 at 104 ms) and to derive a more parsimonious model of non-linear decreases in SSRT with increasing age. Visual inspection of the residuals demonstrated their homogeneity across the model predicted values.

Additive effects on the log-transformed variables were multiplicative in the original (non-log transformed) scale. For this reason, all effects are represented as % difference. Because the effect is expressed as % difference, the corresponding effect in ms will be greater where predictors are associated with larger predicted values (e.g., at younger age) and smaller where predictors are associated with smaller predicted values. For example, a 10.2% greater SSRT in Community ADHD compared to Controls will correspond to a predicted difference of 36 ms at age 6 when the predicted SSRT in Controls is 355 ms but only 21 ms at age 16 when the predicted SSRT in Controls is 207 ms. Raw SSRT and RTV scores values for each group at ages 6, 12 and 16 and effect sizes estimates using the pooled error estimate from the linear regression model are presented in Supplemental Table 3.

We compared each group (ADHD, ASD) to Controls and to each other. We predicted a main effect of group on SSRT and RTV and tested for differences in group trajectories across age, for a total of 8 primary hypotheses: 2 SST outcomes × 2 settings × 2 effects (age x group interaction and group main effect). We use a conservative adjustment for multiple comparisons and α of 0.05/8 = 0.0063. Although this is a very conservative adjustment, results were unchanged even when the more stringent criterion was applied for significance. Where there were no group differences in trajectories across age, the group x age interaction was dropped for a more parsimonious comparison of the main effect of group across all ages.

We did not include differences in RT among our primary hypotheses based on previous findings (Lipszyc & Schachar, 2010). Community models included families with more than one child and therefore we treated family as a random effect (mixed effect linear regression). Models controlled for the effect of age, gender, and stimulant medication (within 24 h prior to testing) if significant. Clinic models were estimated with and without IQ. To estimate the magnitude of impairment, we calculated “age equivalents” (age at which the model predicts a control would perform at the same level) as shown in Supplemental Table 3.

#### Assessing the Impact of Comorbidity

We assessed the effect of ADHD traits in accounting for neurocognitive performance in ASD and ADHD by estimating the proportion of variance in neurocognitive performance that was explained by SWAN t-scores. Our primary interest was the role that ADHD traits play in accounting for neurocognitive performance in ASD. However, these analyses allow us to examine the effect of controlling for ADHD traits while estimating the effect of ADHD as a disorder. Finally, in the Clinic sample, we added SCQ symptom counts to the models to investigate whether ASD symptomatology accounted for any ADHD effects. We did not have an ASD trait measure in the Community sample.

In a secondary analysis, we subdivided the ASD group into those who did/did not meet criteria for a diagnosis of ADHD (6 + inattentive and/or 6 + hyperactive/impulsive symptoms) using a score of 2 or 3 on the SWAN as a symptom (see Supplemental Table 4 for group characteristics). In the Clinic sample this resulted in the following groups: ASD + ADHD (n = 122) and ASD-ADHD (n = 139) and in the Community resulted in the following groups: ASD + ADHD (n = 53) and ASD-ADHD (n = 91). We then reran the SSRT and RTV models (there was no RT effect overall) to compare the ASD group to controls by ADHD classification.

## Results

### Group Characteristics

Table 1 shows age, gender, IQ, SWAN trait t-scores, and SCQ symptom count for each group. The Clinic ADHD group had higher SWAN t-scores than the Community ADHD group suggesting greater severity. The ratio of females to males with ADHD, 1:2.5, was the same in the Clinic and Community ADHD samples, however, the Clinic ASD group included relatively more females (1:3.7 F:M ratio) than did the Community ASD group (1:6.3) (Table 1). Supplemental Fig. 1 and Supplemental Table 1 show medication use in the various groups. Stimulant medication use was controlled for in the analyses below. In the community sample, comorbid anxiety was reported in 2% of controls, 19% of ASD, 14% of ADHD and 7% of the High Trait group. Comorbid depression was reported in < 1% of controls, 3% of ASD, 3% of ADHD and 4% of the High Trait group. Comorbid anxiety was reported in 21% of ASD and 21% of ADHD clinic participants and in none of the clinic controls. We did not have information on comorbid depression in the clinic sample.

**Table 1:** Clinical characteristics of ADHD, ASD and Control groups in Clinic and Community

			**Age**	**SWAN** **t-score**^**b**^	**SCQ Symptom Count**^*c*^	**IQ**
**Clinic**		*n (%)*	*Mean (sd)*	*Mean (sd)*	*Median (IQR)*^*d*^	*Mean (sd)*
Controls	Males	99 (65)	11.7 (2.8)	44.8 (10.4)	2 (1;4)	108.6 (12.6)
	Females	63 (35)	11.7 (3.1)	43.2 (10.2)	2 (1;3)	107.7 (13.1)
ADHD	Males	301 (71)	10.4 (2.8)	66.7 (7.4)	5.2 (3;10)	100.9 (15.1)
	Females	122 (29)	9.9 (2.4)	68.5 (7.2)	5 (2;9)	98.6 (15.8)
ASD	Males	205 (79)	11.7 (3.1)	63.3 (8.8)	17 (13;22)	95.0 (19.2)
	Females	56 (21)	12.3 (3.0)	64.3 (8.6)	18 (10,21.6)	97.3 (16.6)

**Community**						
Controls	Males	7,182 (48)	10.8 (2.7)	48.1 (8.8)		
	Females	7,660 (52)	11.2 (2.9)	49.2 (8.8)		
ADHD	Males	663 (72)	11.3 (2.6)	61.7 (8.3)		
	Females	263 (28)	12.0 (2.7)	63.6 (9.1)		
ASD	Males	164 (86)	11.0 (2.4)	61.7 (8.3)		
	Females	26 (14)	10.9 (2.8)	64.7 (7.3)		
ADHD High Trait^a^	Males	207 (61)	10.6 (2.8)	67.8 (5.6)		
	Females	130 (39)	12.1 (3.3)	70.7 (5.9)		

^a^*High Trait* SWAN total, inattentive, and/or hyperactive t-score > 70 but no community diagnosis of ADHD or ASD

^b^SWAN t-scores do not control for gender

^c^SCQ symptom count is sum of items coded as yes

^d^*IQR* Interquartile Range

### Neurocognitive Performance

#### Response Inhibition (SSRT) in Clinic Groups

There was a significant main effect of group on SSRT (*F*(2,757) = 13.56, p < 0.0001). SSRT was 18.8% longer in the Clinic ADHD group than in the Clinic Control group and 23.1% longer in the Clinic ASD group than in the Clinic Control group (group scores, % difference, 95% CI and *p* values are shown in Table 2 (raw scores, age equivalents and effect sizes in Supplemental Table 3).

**Table 2:** Effect of disorder on SSRT in community and clinic samples

	**Clinic**		**Community**	
	*% difference (95% CI)*	*p*	*% difference (95% CI)*	*p*
**Disorder Group**	***F*2,757) = 13.56**	**< 0.0001**	***F*(3, 2952) = 14.12**	** < 0.0001**
ADHD vs Controls	18.8 (10.1,28.2)	< 0.0001	10.2 (5.7,14.9)	< 0.0001
ASD vs Controls	23.1 (13.3,33.7)	< 0.0001	11.2 (2.4, 20.7)	0.012
ASD vs ADHD	3.6 (-3.2,10.9)	0.3	0.9 (-7.6,10.3)	0.8
**Disorder Group**	**F(2,749) = 2.47**	**0.09**	**F(3,2951) = 1.55**	**0.2**
** + SWAN t-score**	**F(1,749) = 11.54**	**0.0007**	**F(1,2951) = 102.34**	** < 0.0001**
ADHD vs Controls	5.3 (-5.4,17.2)	0.3	3.7 (-0.7,8.3)	0.1
ASD vs Controls	11.1 (0.1,23.2)	0.048	5.4 (-3.0,14.5)	0.2

Clinic models control for Disorder (ADHD, ASD and Controls), age, stimulant medication and an age x stimulant medication interaction (upper part of table) + SWAN t-score (lower part of table); Community models control for Disorder (ADHD, ASD, High Trait and Controls), age, age^2^, gender and stimulant medication (upper part of table) + SWAN t-score (lower part of table)

When SWAN t-scores were added to the models to control for ADHD trait severity across ADHD and ASD, the effect of SWAN t-score was significant but the effect of disorder was no longer significant (group p = 0.09). Controlling for SWAN t-scores reduced the Clinic ASD effect by 52% (Clinic ASD effect p = 0.048). Given that ADHD is essentially “defined” by the presence of ADHD symptoms it was not surprising that controlling for SWAN t-scores reduced the Clinic ADHD group effect by 72% confirming that the effect of ADHD on SSRT was largely attributable to ADHD trait severity. SCQ symptom count was not significant when added to the models indicating that ASD traits did not account for poorer response inhibition in the Clinic ADHD group (data not shown).

**Fig. 1:**
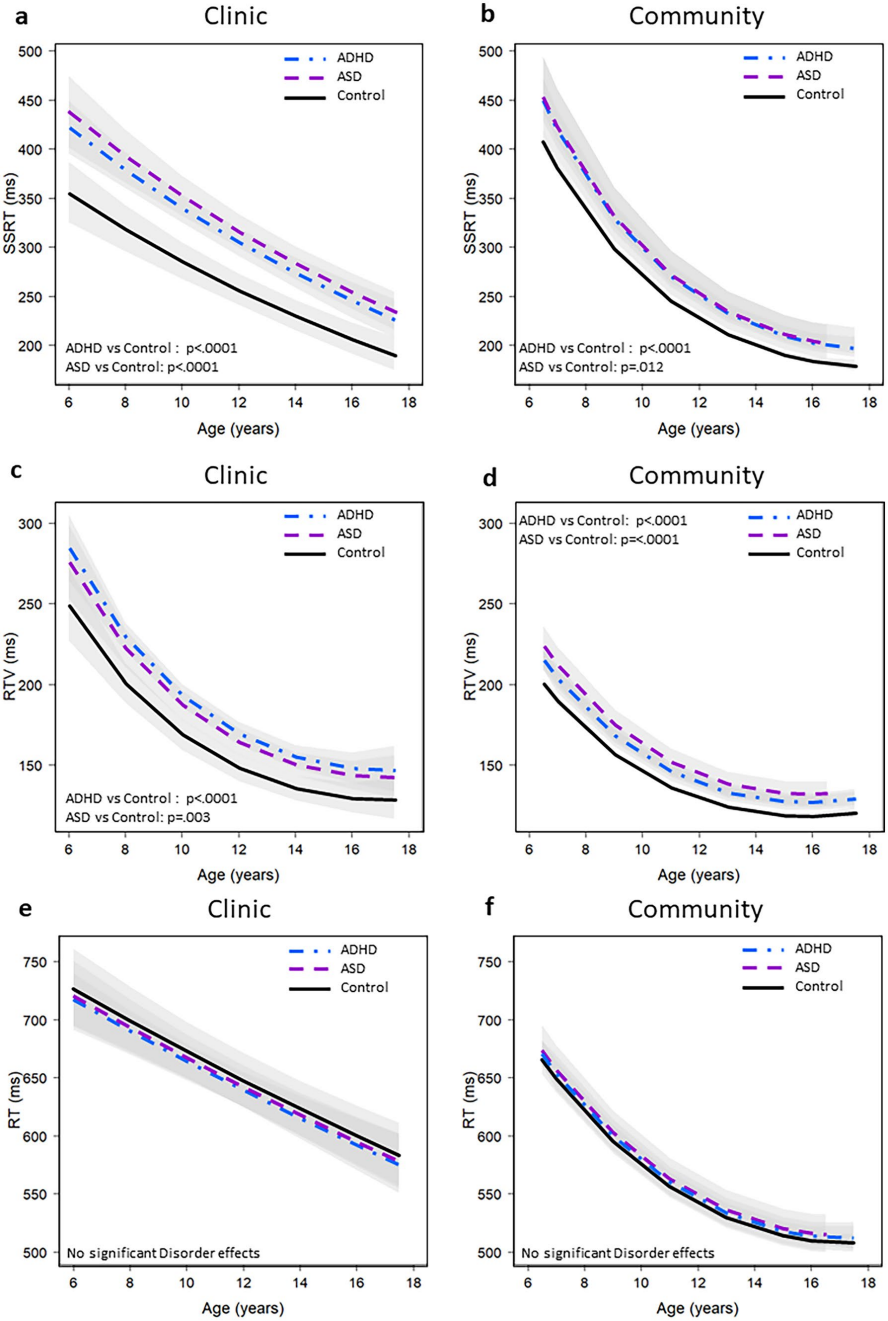
Stop Signal Task performance in Clinic and Community samples. Distribution of stop-signal reaction time (SSRT – Panel **A** & **B**), reaction time variability (RTV – Panel **C** & **D**) and reaction time (RT – Panel **E** & **F**). Clinic samples are presented on the left side (Figure **a** ASD: n= 233, ADHD: n=371, Controls: n=159 and **c; e** ASD: n=261, ADHD: n=423, Controls: n=162) and community samples are presented on the right side (Figure **b** ASD: n=123, ADHD: n=698, Controls: n=11,428) and **d, f **ASD: n=144, ADHD: n=749, Controls: n= 12,095). The bands around each line represent the 95% confidence interval. These values are not corrected for comorbidity

There was a clear age effect but no significant group difference by age effect (Supplemental Table 5) on SSRT (Fig. 1a). In the Clinic sample, IQ was not significant when added to the model with group and SWAN t-score (*F* = 2.89, *p* = 0.09). A 10-point difference in IQ was associated with a 2.0% lower SSRT in the clinic sample in a model that does not control for SWAN (95% CI: -3.8, -0.01%, p = 0.035). Controlling for IQ did not have any impact on the ASD and ADHD differences in SSRT from controls.

Table 3 shows the comparison of SSRT in the Clinic ASD + ADHD with ASD-ADHD groups and Supplemental Table 4 shows the characteristics of ASD – ADHD, ASD + ADHD, and Control groups in Clinic and Community SSRT and RTV models. The Clinic ASD + ADHD group had significantly longer SSRT than the ASD-ADHD group. Both the Clinic ASD + ADHD and ASD-ADHD groups had significantly longer SSRT than the Clinic control group and the ASD + ADHD group had significantly longer SSRT than the ASD-ADHD group.

**Table 3:** SSRT and RTV in ASD + ADHD and ASD-ADHD in Clinic and Community samples

	**Clinic**		**Community**	
**SSRT**	*% difference (95% CI)*	*p*	*% difference (95% CI)*	*p*
**ASD vs Control**				
- ADHD	14.2 (4.0,25.4)	0.006	3.6 (-6.5,14.7)	0.5
+ ADHD	35.9 (22.8,50.3)	< 0.0001	26.1 (10.1,44.4)	0.0008
ASD + vs–ADHD	19.0 (7.2,32.0)	0.001	21.8 (2.9,44.0)	0.022

**RTV**	*% difference (95% CI)*	*p*	*% difference (95% CI)*	*p*
**ASD vs Control**				
- ADHD	7.7 (-0.3,16.3)	0.058	3.7 (-3.0,11.0)	0.3
+ ADHD	14.9 (5.9,24.7)	0.0009	25.4 (15.0,36.8)	< 0.0001
ASD + vs–ADHD	6.7 (-1.9,15.9)	0.1	20.9 (8.4,34.9)	0.0007

#### Response Inhibition (SSRT) in Community Groups

There was a significant main effect of group on SSRT in the community sample *(F*(3, 2952) = 14.12, *p* < 0.0001). As shown in Table 2 and Supplemental Table 3, SSRT was 10.2% longer in the Community ADHD group and 11.2% longer in the Community ASD group than in the Community Control group. Controlling for SWAN t-scores eliminated the effect of group and reduced the ASD effect by 52% (Table 2). The Community ASD + ADHD group, but not the ASD-ADHD group had significantly longer SSRT than did the Community control group (Table 3). The High Trait group had significantly longer SSRT than controls (13.9% longer) and did not differ from ASD or ADHD groups (see Supplemental Table 6).

The magnitude of the SSRT impairment in the Community Disorder groups was about half as great as that in the Clinic groups suggesting that severity might be lower in the Community sample. There was a clear effect of age which did not differ significantly across groups in the Community sample (Fig. 1b, Supplemental Table 5).

#### Reaction Time Variability (RTV) in Clinic Sample

There was a significant main effect of group on RTV in the Clinic sample (*F*(2,840) = 8.77, *p* = 0.0002) with no group differences in the age effect (Supplemental Table 5). RTV was 14.3% greater in the Clinic ADHD group and 10.8% greater in the Clinic ASD group than in the Clinic Controls (Table 4, Fig. 1c and Supplemental Table 2). Controlling for SWAN t-scores reduced the RTV deficit in the Clinic ASD group by 73% and was no longer significant. SCQ symptom count was not significant when added to models with group (*F* = 2.70, *p* = 0.1) indicating that ASD traits did not impact the performance of the ADHD group. The Clinic ASD + ADHD group had greater RTV than controls (14.9% greater), but the difference between Clinic ASD-ADHD and Control groups (7.7%) was not significant (Table 3). IQ was significant when added to the model with Disorder and SWAN t-score (*F* = 5.50, *p* = 0.019), but did not change the results of the group comparisons. A 10-point difference in IQ was associated with 2.0% lower RTV in the clinic sample (95% CI: -3.4, -0.5%, p = 0.007). Controlling for IQ reduced but did not eliminate the ASD and ADHD differences from controls.

**Table 4:** Effect of disorder on RTV in community and clinic samples^1^

	**Clinic**		**Community**	
	*% difference (95% CI)*	*p*	*% difference (95% CI)*	*p*
**Disorder Group**	***F*(2,840) = 8.77**	**0.0002**	***F*(3,3279) = 18.24**	**< 0.0001**
ADHD vs Controls	14.3 (7.4,21.7)	< 0.0001	7.2 (4.6,9.8)	< 0.0001
ASD vs Controls	10.8 (3.6,18.6)	0.003	11.4 (5.5,17.5)	< 0.0001
ASD vs ADHD	3.1 (-2.3,8.8)	0.3	3.9 (-1.9,10.2)	0.2
**Disorder Group**	**F(2,831) = 0.51**	**0.6**	**F(3,3278) = 2.69**	**0.045**
**+ SWAN t-score**	**F(1,831) = 8.77**	**0.003**	**F(1,3278) = 115.21**	**< 0.0001**
ADHD vs Controls	4.5 (-4.3,14.1)	0.3	2.2 (-.4,4.9)	0.1
ASD vs Controls	2.9 (-5.5,12.1)	0.5	6.8 (1.2,12.8)	0.017

Clinic models control for Disorder (ADHD, ASD and Controls), age, age^2^, and stimulant medication (upper part of table) + SWAN t-score (lower part of table); Community models control for Disorder (ADHD, ASD, High Trait and Controls), age, age^2^, gender and its interaction with age and age^2^ (upper part of table) + SWAN t-score (lower part of table)

#### Reaction Time Variability in Community Sample

There was a significant main effect of group on RTV in the Community sample (*F*(3,3279) = 18.24, *p* < 0.0001). RTV was 7.2% greater in the Community ADHD group and 11.4% greater in the Community ASD group than in the Community Controls (Table 4, Fig. 1d, and Supplemental Table 3). Controlling for SWAN t-scores had a significant effect on RTV so that the disorder effect no longer met the multiple comparison adjusted threshold for significance (p = 0.045), despite the fact that control for SWAN t-scores only reduced the Community ASD effect by 40% (Community ASD effect p = 0.017). The magnitude of the RTV effect in the Community ADHD group was about half as great as that of the Clinic groups suggesting an effect of ADHD trait severity consistent with lower SWAN t-scores in the ADHD Community sample than in the Clinic sample. The effect of ADHD trait severity was confirmed by the finding that RTV was significantly greater in the Community ADHD High Trait (7.1%) than in Controls (Supplemental Table 6). By contrast, the magnitude of the effect in Community and Clinic ASD was about the same. There was a significant effect of age on RTV in the community sample that did not vary across group (Supplemental Table 5).

#### Reaction Time in Community and Clinic Samples

There was no group difference in either the Clinic or Community samples for RT (Fig. 1e-f).

#### Estimation of the Magnitude of the Observed Effects

Estimation of age equivalence showed that ADHD and ASD groups were delayed in achieving control levels of performance in SSRT and in RTV by about 1 to 4 years (Supplemental Table 3). For example, a Clinic 12-year-old with ADHD not using stimulant medication is predicted to have an SSRT of 305 ms. A control would be expected to have an SSRT of 305 ms at the age of 8.8 years, equivalent to a delay in neurocognitive development of over 3 years.

#### Gender, Medication, and Age Effects in Clinic and Community Samples

Females had 4.4% longer SSRT than males [(2.7,6.1), *p* < 0.0001) in the Community but there was no gender effect in the Clinic sample. There were no significant gender x diagnosis interactions in any of the models (Supplemental Table 5). There was no interaction of SWAN and disorder across any of the models (Supplemental Table 5) which means the effect of SWAN was the same in the lower range (Control group) and upper range of both ADHD and ASD. Current stimulant medication use was associated with a 7.9% shorter SSRT across all ages in the Community sample [(-14.0,-1.3), *p* = 0.019] and with shorter SSRT at younger but not older ages in the Clinic sample. Stimulant medication was associated with a reduction in RTV of 9.3% [(-15.6,-2.6), *p* = 0.007] in the Clinic sample but was not significant in the Community sample. Figure 1 shows that SSRT and RT were faster and RTV was less with increasing age; however, age did not alter the group effects described above. Correlation between RTV and SSRT was low ranging from 0.07 in the TD group to 0.28 in the community ASD group (see Supplemental Table 7). Correlation between SSRT and RTV was higher in younger than older participants and among the community than in clinic participants (see Supplemental Table 7).

## Discussion

We compared ADHD and ASD cases with controls on two indices of neurocognition (SSRT and RTV) with and without control for comorbid ADHD and ASD to determine whether ADHD and ASD share neurocognitive profiles. We conducted these comparisons in specialty clinics where ADHD and ASD diagnosis was established after rigorous assessment and in a community sample where ADHD and ASD was defined by parent and self-report in order to assess the generalizability of findings. This study was motivated by the fact that there have been few direct comparisons of neurocognitive function, in particular response inhibition, in large samples of individuals with ADHD and ASD (Albajara Sáenz et al., 2020; Kuijper et al., 2015, 2017; Van Hulst et al., 2018). We focused on two key neuropsychological processes. SSRT is a well-established measure of response inhibition–the speed with which one can stop a speeded motor response. RTV has been interpreted in various ways, but the strongest case can be made for it to be a reflection of lapses in attention (Kofler et al., 2013).

The current study more than doubled the number of ASD and ADHD participants in existing comparative studies. Moreover, there has been only one comparative study in which ADHD comorbidity has been controlled (Karalunas et al., 2018). To assess the impact of ADHD comorbidity on ASD, we examined neurocognitive function in ASD with and without control for continuously measured ADHD traits using the SWAN rating scale–a normed and valid measure of ADHD traits as well as compared ASD with and without categorically defined comorbid ADHD in both clinic and community samples. We additionally checked for the impact of ASD comorbidity as measured by social cognitive deficits on neuropsychological performance, in ADHD using SCQ symptom counts in the clinic sample (c.f., Karalunas et al., 2018).

Without control for ADHD traits, *both* Clinic and Community ADHD and ASD groups showed longer (impaired) response inhibition (SSRT) and greater reaction time variability (RTV) than age-matched controls, but did not differ in response time (RT). The differences in SSRT and RTV between ASD and ADHD were not significant. Because it can be difficult to estimate the clinical significance of differences in reaction time measures, we estimated the magnitude of the impairments using “age equivalents''–the age at which statistical models predicted that a control would perform at the same level as a case. The difference between ADHD, ASD and controls was substantial. Clinic ADHD and Clinic ASD showed a 2-year delay in SSRT. For RTV, the delay was 1–2 years. The delay for Community cases was somewhat less for SSRT but similar for RTV. The functional implications of this effect needs further study as measures of impairment were not collected, but we note that a 2-year delay is the typical criterion for learning disability. We caution that while the observed impairments were statistically significant and may be clinically or aetiologically relevant, the associations were not sufficiently strong or sufficiently specific to use as a proxy for diagnosis (Zakzanis, 2001).

In a large direct comparison of ASD and ADHD, Karalunas et al. (2018) reached the same conclusion as this study regarding impaired SSRT and greater RTV in ADHD and ASD. However, our conclusions about the role of comorbid ADHD differ from those of Karalunas and colleagues. We found that comorbid ADHD explained the deficits in ASD. We came to this conclusion after assessing the role of ADHD comorbidity in several ways. We added continuous trait scores for ADHD to the SSRT models and found that the effect of diagnosis on SSRT and RTV were no longer significant in either clinic or community samples, but the effect of ADHD trait severity was significant. In the case of ADHD, this result is not surprising. ADHD is a disorder defined by ADHD traits. After control for ADHD traits, the ASD group no longer exhibited a longer SSRT than controls in the community sample and the difference from controls in the clinic sample was not significant after correction for multiple testing. The story was similar with respect to RTV. Controlling for ADHD traits reduced the difference in ASD versus controls to a non-significant level in the clinic sample and reduced but did not eliminate the effect of ASD in the community sample. In contrast, Karalunas et al. (2018) found no effect of ADHD traits on neurocognitive performance in the ASD group. The Karalunas et al. (2018) and current study differ in a number of important ways. Karalunas used the ADHD rating scale (DuPaul et al., 1998) as their measure of ADHD traits. The ADHD rating scale is truncated at zero where zero indicates that a trait is not present. By contrast, the SWAN allows for ratings from strengths to weaknesses so that zero indicates average behavior and minus scores reflect strengths. Using the ADHD Rating Scale could result in a loss of power to detect ADHD trait effects in their control sample compared to an analysis that uses the SWAN. Karalunas also dropped the ADHD group from their analyses controlling for ADHD traits. This would further cause a loss of power to detect an ADHD trait effect. We assume that they were concerned with picking up an ADHD trait effect driven by the ADHD sample that did not hold in the ASD sample. We checked this possibility by testing for a trait by group interaction and found no evidence that the ADHD trait effect was different in the ASD sample than in the control or ADHD samples. It should be noted that Karalunas et al. calculated RTV using all trials for which there was a response including those that followed unsuccessful efforts to stop. These responses are largely slower than trials for which stopping was not required (Dupuis et al., 2018). We think that slowing after successful and unsuccessful stopping should be distinguished from RTV because of the potential confound between number of failed efforts to stop and RTV.

Karalunas et al. (2018) found that the ASD-ADHD group was like the ASD + ADHD group and more impaired than controls. From this, they concluded that ADHD did not explain the effect of ASD. By comparison, we found the opposite-the ASD-ADHD group tended to align more closely with controls than with the ASD + ADHD group. Unlike Karalunas, we attribute any residual ASD-ADHD effect in the clinic to the large difference between the ASD-ADHD and Control groups in SWAN t-score even though this group did not include participants with comorbid ADHD. The difference in SWAN scores was larger in the clinic sample (14.4%) than in the community sample (8.2%) which may explain why the ASD-ADHD effect was significant in the clinic but not the community sample. A closer look at the Supplemental Table in Karalunas demonstrates that the total ADHD Rating Scale score for the ASD-ADHD was a full standard deviation greater than for controls. Looked at together, we conclude that a large proportion of the effect in ASD is driven by elevated ADHD traits with some much smaller proportion of variance driven by ASD specific deficits.

The current conclusion that response inhibition and reaction time variability impairment in ASD is largely, although not exclusively, associated with comorbid ADHD traits is consistent with other studies (Corbett et al., 2009; Happé et al., 2006; Rommelse et al., 2010; Salunkhe et al., 2021; Tye et al., 2016). A recent meta-analysis of research in ASD identified 42 studies of inhibition which included 1534 participants (Lai et al., 2017). But only 11 of these studies, involving 242 participants, controlled for ADHD. Similar to the current results, the magnitude of the deficit in ASD was reduced with control for ADHD (Lai et al., 2017).

These results highlight the strong link between ADHD traits and neurocognitive test performance across disorders. This effect is most obvious in the strong and replicated deficits that are found in ADHD per se. The association of ADHD trait severity and neurocognition is also supported by the significant role of ADHD traits in predicting SSRT and RTV and in the neurocognitive impairments that we observed in the community high trait groups which reported neither ADHD nor ASD. Moreover, the association of ADHD traits and neurocognitive deficit, at least insofar as it was measured in this study, appears to be similar in ASD as in ADHD. More generally speaking, it appears as if ADHD traits are indicators of neurocognitive impairment in whatever disorder they are found. If one controls comorbid ADHD, neurocognitive impairment is essentially eliminated. You can see this in the fact that impaired response inhibition is found in anxious children with comorbid ADHD traits but not in those without comorbid ADHD (Korenblum et al., 2007). Studies of other neurocognitive processes and variants of inhibition are needed to determine the generality of this conclusion.

Response inhibition and reaction time variability might prove to be good, cross-disorder markers of aetiological risk factors in disorders characterized by ADHD traits given that both response inhibition and RTV have genetic (Finkel & Pedersen, 2014; Friedman et al., 2008; Schachar et al., 2005) and neurobiological (Albaugh et al., 2017; Chevrier & Schachar, 2020; Sonuga-Barke & Castellanos, 2007) underpinnings. We observed low correlation between RTV and SSRT across community and clinic groups among older and younger participants suggesting that these performance indices reflect separable rather than common processes (cf Karr et al., 2018). These results also indicate that the ADHD associated with ASD is not a phenocopy of “true” ADHD at least using neurocognitive impairment as the criterion for phenocopies. As has been found in many previous studies, comorbid ADHD was common in ASD in both clinic and community ASD samples (Brookman-Frazee et al., 2018; Joshi et al., 2017; Lyall et al., 2017).

Several other findings that emerged from the current study are worthy of mention. Differences among disorders in SSRT and RTV did not vary with age although age did affect performance as has been found previously (Crosbie et al., 2013). The fact that there was no interaction between SWAN and disorder indicates that the effect of ADHD traits was the same in the lower range (among controls) as it was at the upper range, providing additional support for the quantitative nature of the ADHD effect. By contrast, social cognition as measured with SCQ symptom counts did not significantly impact SSRT or RTV when added to disorder in the models, indicating that ASD traits did not affect neurocognitive function in ADHD (or ASD for that matter) over and above the effect of diagnosis and ADHD trait severity. It is possible that the SCQ is less sensitive to ASD-related quantitative traits than the SWAN questionnaire because it lacks the capacity to measure the full spectrum of ASD traits.

IQ differences between ASD and ADHD or controls did not explain the observed impairments in neurocognitive test performance (Dennis et al., 2009). Nor were neurocognitive differences between ADHD and ASD a function of stimulant medication taken around the time of testing. ADHD medication was widely used in ADHD and ASD participants. Control for stimulant usage was important because it predicted 7.9% shorter SSRT across all ages in the Community sample and shorter SSRT at younger but not older ages in the Clinic sample.

We found no support for the contention that longer SSRT or greater RTV were artifacts of slower response times as previously asserted (Alderson et al., 2008; Huang-Pollock et al., 2012). The tracking algorithm in the stop task is designed to separate reaction time from response inhibition (see Supplemental material for details). However, reaction time is a complex process that can be operationalized in various ways (Rommelse et al., 2020). In this study, we examined the speed of responding in a choice reaction time task where participants are required to make one of several different responses depending on which one of several stimuli are presented (respond with one hand if you see an ‘X’ and with the other hand if you see an ‘O’), namely reaction to the go stimuli in trials that did not involve stop signals. Previous research supports the hypothesis that processing speed might be slower in ADHD when operationalized as performance in the coding and symbol search subscales of intelligence tests (Braaten et al., 2020; Nigg et al., 2017).

## Limitations

This study lacked an independent measure of impairment against which to judge the clinical significance of observed neurocognitive deficits. Direct comparison of ADHD and ASD on other neurocognitive functions is necessary to fully identify the executive function deficits and strengths of each diagnosis (c.f., Carter Leno et al., 2018; Tye et al., 2016). An association between neurocognitive impairment and ADHD does not prove that the neurocognitive deficit is the cause of the disorder. That question could be addressed through cross-sectional and longitudinal mediation and moderation analyses of genetic, neurocognitive function and ADHD traits or diagnosis once clear genetic risks are established.

Both the community and clinic samples are likely to be shaped by hidden biases. Fewer severely affected individuals might come to the science museum because of the noisy and distracting environment or might not be willing to participate in a study that required testing on a computer task of attention. Referral to specialty clinics and recruitment into clinical research might be biased toward greater severity of comorbid conditions (e.g., anxiety and depression) that we did not measure. The greater number of invalid SST administrations in the ADHD and ASD sample means those with the worst performers were excluded at a greater rate in those groups than in the control group, resulting in underestimation of the true magnitude of the difference between the groups.

## Conclusions

These results support the conclusion that ADHD and ASD share a neurocognitive profile characterized by deficient response inhibition and sustained attention reflected in greater reaction time variability but not RT. ADHD traits and/or comorbidity accounted for the observed impairment in ASD.

## Supplementary Information

Below is the link to the electronic supplementary material.

Supplementary file1 (DOCX 120 KB)
