# Supplementary material for: Autism Spectrum Disorder and Attention-Deficit/Hyperactivity Disorder: Shared or Unique Neurocognitive Profiles?
Source: Res Child Adolesc Psychopathol. 2022 Aug 25;51(1):17–31. doi: 10.1007/s10802-022-00958-6 (PMC9763138; doi:10.1007/s10802-022-00958-6)
Supplement: Supplementary file 1 — Supplementary file1 (DOCX 120 KB) [file 10802_2022_958_MOESM1_ESM.docx]

**Supplemental materials**

**Methods**

1. **SWAN standardized scores**

We measured ADHD traits using the Strengths and Weaknesses of ADHD Symptoms and Normal Behavior Scale (SWAN). SWAN covers the previous 6 months. We calculated standardized scores taking age and respondent into account. Linear regression was used to model SWAN total scores controlling for age and age^2^. We had both parent- and self-reports on a subset of participants and found no effect of respondent on SWAN scores. Therefore, respondent (self vs parent) was not controlled for in the SWAN models. In order to remove heteroscedasticity (in particular, higher variance at younger ages), we used linear regression to model the residuals controlling for age and age^2^, further adjusting for skewness by modeling negative and positive residuals separately. The predicted values from this model provided an estimate of the standard deviation of the data for a given age. Normative t-scores were derived by dividing individual residuals by their predicted standard deviation. Minor adjustments to the final scores were made to ensure that each set of scores had an overall mean of 50 and standard deviation of 10.

1. **Stop signal task (SST)**

The SST (simple stop task) was presented on a notebook computer using Presentation software to control timing of stimuli and a gamepad for responding. The SST consists of two tasks: Go task and stop task. The go task involves responding to either an X or an O presented in the middle of the screen for 500 ms following a fixation lasting 500 ms. Participants were instructed to respond to the go stimuli with a right-hand response if an X appeared on the screen or a left-hand response if an O appeared and to do so as fast as possible without making mistakes. The stop task involved presentation of a stop signal through over-the-ear headphones at a comfortable listening volume, randomly on 25% of go trials. The stop signal was initially presented 250 ms after the go signal and dynamically adjusted depending on performance. Participants were instructed to stop their response if they could when they heard the stop signal. If a response were stopped, the stop signal delay increased by 50 ms making it more difficult to stop on the next trial. If they were unable to stop, stop-signal delay decreased by 50 ms making it easier. The task consisted of a practice block (24 trials; 18 go trials; six stop trials) and four experimental blocks of 24 trials for a total of 72 go trials and 24 stop trials.

Stop-signal reaction time was calculated as the difference between mean go RT on go trials (no signal trials) and mean delay using interpolation method.

We took various precautions to ensure consistency of administration across sites and over time. First, we wrote and debugged the software for the SST over many years. Second, we ensured that the software could run properly on every computer and that every computer had the necessary specifications and set up (e.g. encryption software disabled). Third, we collected all results, both scored and raw data files, at a central location where we could check the validity of every administration and identify any computers that were generating poor data. As described in the manuscript, some administrations were excluded because of invalid performance (usually low accuracy on go task, many pre-pushes or no-response trials). Finally, our programme included built-in instructions for participants. Participants were presented with an image of the button box indicating which keys to push and were asked to demonstrate their ability to do so. The practice software monitored accuracy of responses. Then the task was introduced step by step to ensure that participants were performing it correctly. Participants could ask for help if they did not understand task instructions. Between task blocks, performance was presented on the screen to enable the supervising research staff to note failures of administration e.g., participants were going slower and slower, failing to respond, pushing the wrong buttons for X and O etc. These interim results were presented in such a way as to ensure that participants were not getting feedback. Our version of the SST is available for other researchers by emailing the corresponding author.

**Supplemental Results**

**Supplemental Figure 1: Medication use in community and clinic participants – n (%)**

Note. Stim/Non stim = stimulant (e.g., methylphenidate) and non-stimulant (atomoxetine) medications typically used in treatment of ADHD. Participants using both non-stimulant and stimulant medications for ADHD are classified as taking stimulant medications for the purpose of this figure. SSRI=specific serotonin reuptake inhibitors typically used in treatment of depression, anxiety, and OCD.

**
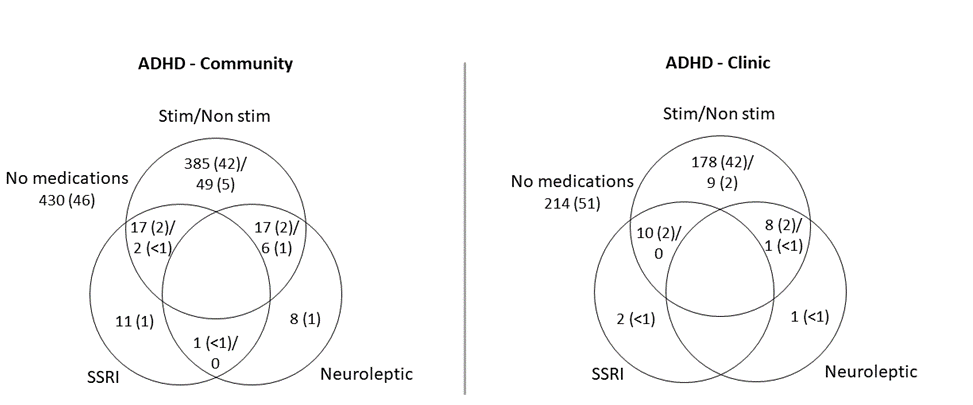
**

**
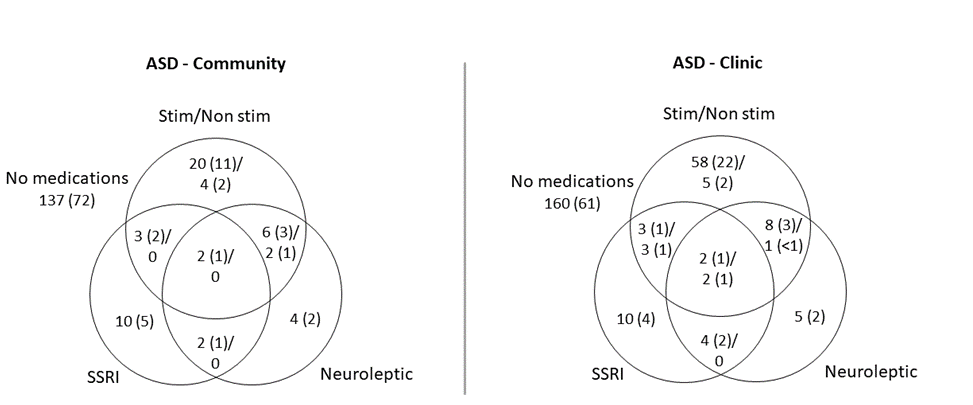
**

**Supplemental Table 1: Type of medication recently used by ADHD, ASD and High Trait participants in Clinic and Community samples.**

|  |  | **SSRI** | **ADHD medication** | **Neuroleptic** |
| --- | --- | --- | --- | --- |
|  | ***n*** | ***n (%)*** | ***n (%)*** | ***n (%)*** |
| **ADHD** |  |  |  |  |
| **Clinic** | 423 | 12 (2.8) | 206 (48.7) | 10 (2.4) |
| **Community** | 926 | 31 (3.3) | 476 (51.4) | 32 (3.5) |
| **ASD** |  |  |  |  |
| **Clinic** | 261 | 24 (9.2) | 82 (31.4) | 22 (8.4) |
| **Community** | 190 | 17 (8.9) | 37 (19.5) | 16 (8.4) |
| **High ADHD Trait** |  |  |  |  |
| **Community** | 337 | 3 (0.9) | 0 | 1 (0.3) |

**Supplemental Table 2: SST Consort table**

| **Community** | **TD** | **ADHD** | **ASD** | **High trait** |
| --- | --- | --- | --- | --- |
| Sample who attempted SST | 12,104 | 750 | 144 | 265 |
| **SSRT validity screen:** |  |  |  |  |
| % stop inhibit <15 or >85 | 254 | 19 | 7 | 9 |
| > 1/8 no responses | 260 | 26 | 9 | 16 |
| > 2 stop pre pushes | 120 | 2 | 4 | 3 |
| SSRT < 50ms | 42 | 5 | 1 | 3 |
| **Total SSRT exclusions (%)** | **676 (5.6)** | **52 (6.9)** | **21 (14.6)** | **31 (11.7)** |
| *Odds Ratio (vs TD), 95%CI* |  | 1.5 (1.1,2.0) | 3.2 (2.0,5.2) | 2.4 (1.6,3.5) |
| *p* |  | .011 | <.0001 | <.0001 |
| Valid SSRT | 11,428 | 698 | 123 | 234 |
| Valid RT and RTV^1^ | 12,095 | 749 | 144 | 264 |
| **Clinic** | **TD** | **ADHD** | **ASD** |  |
| Sample with valid stop | 162 | 423 | 261 |  |
| **SSRT validity screen:** |  |  |  |  |
| % stop inhibit <15 or >85 | 2 | 12 | 12 |  |
| > 1/8 no responses | 1 | 26 | 11 |  |
| > 2 stop pre pushes | 0 | 8 | 4 |  |
| SSRT < 50ms | 0 | 6 | 1 |  |
| **Total SSRT exclusions (%)** | **3 (1.9)** | **52 (12.3)** | **28 (10.7)** |  |
| *Odds Ratio (vs TD), 95%CI* |  | 5.6 (1.7,18.4) | 6.7 (2.0,22.7) |  |
| *p* |  | .004 | .002 |  |
| Valid SSRT | 159 | 371 | 233 |  |
| Valid RT and RTV^1^ | 162 | 423 | 261 |  |

^1^ a minimum of n=5 trials are needed to estimate post correct go RT and RTV

**Supplemental Table 3:** Predicted raw scores, age equivalents and effect size for SSRT and RTV at ages 8, 12 and 16 for each group.

|  |  | **Clinic Predicted value (ms)**  **(95% CI) at age:** | | | **Community Predicted value (ms)**  **(95% CI) at age:** | | |
| --- | --- | --- | --- | --- | --- | --- | --- |
|  |  | **8** | **12** | **16** | **8** | **12** | **16** |
| SSRT | Control | 318 (296,343) | 286 (268,305) | 207 (191,223) | 335 (331,340) | 227 (224,229) | 185 (181,188) |
|  | ADHD | 378 (360,397) | 305 (291,319) | 246 (228,264) | 369 (354,386) | 250 (240,260) | 204 (195,213) |
|  | ASD | 392 (366,420) | 316 (299,333) | 255 (237,273) | 373 (343,405) | 252 (232,274) | 205 (189,223) |
|  | ADHD High Trait |  |  |  | 382 (360,405) | 258 (243,274) | 210 (198,223) |
| RTV | Control | 200 (189,213) | 148 (140,157) | 130 (121,139) | 172 (170,173) | 129 (128,130) | 119 (117,120) |
|  | ADHD | 229 (221,238) | 169 (162,177) | 148 (139,158) | 184 (179,188) | 139 (135,142) | 127 (124,131) |
|  | ASD | 222 (211,234) | 164 (156,173) | 144 (135,153) | 191 (181,202) | 144 (137,152) | 132 (125,140) |
|  | ADHD High Trait |  |  |  | 184 (179,188) | 139 (133,144) | 127 (122,132) |
|  |  | **Clinic Control age equivalence^1^** | | | **Community Control age equivalence^1^** | | |
| SSRT | ADHD | *< 6.0* | 8.8 | 12.8 | 7.2 | 10.8 | 13.7 |
|  | ASD | *< 6.0* | 8.1 | 12.1 | 7.2 | 10.7 | 13.5 |
|  | ADHD High Trait |  |  |  | 7.0 | 10.4 | 13.1 |
| RTV | ADHD | 6.7 | 9.9 | 12.0 | 7.3 | 10.7 | 12.4 |
|  | ASD | 7.0 | 10.4 | 12.6 | 6.9 | 10.1 | 11.6 |
|  | ADHD High Trait |  |  |  | 7.3 | 10.7 | 12.4 |
|  |  | **Clinic Effect Size^2^** | | | **Community Effect Size^2^** | | |
| SSRT | ADHD | 0.43 | | | 0.25 | | |
|  | ASD | 0.52 | | | 0.23 | | |
|  | ADHD High Trait |  | | | 0.31 | | |
| RTV | ADHD | 0.39 | | | 0.36 | | |
|  | ASD | 0.31 | | | 0.23 | | |
|  | ADHD High Trait |  | | | 0.23 | | |

^1^ The age at which the model predicts a control would perform at the same level. For example, a clinic 12-year-old with ADHD not using stimulant medication is predicted to have an SSRT of 305 ms. A control would be expected to have an SSRT of 305 ms at the age of 8.8 years; where the predicted age value falls outside the age range in our sample, it is shown as < 6.0.

^2^ Effect size estimates use the pooled error estimate from the linear regression model (correcting for other variables in the model) on the log-transformed outcome: Clinic SSRT: 0.398; Clinic RTV: 0.336; Community SSRT: 0.454; Community RTV: 0.300

**Supplemental Table 4:** Characteristics of ASD – ADHD, ASD + ADHD, and Control groups in Clinic and Community SSRT and RTV models**.**

|  |  | **Males** | **Age** | **SWAN t-score** |
| --- | --- | --- | --- | --- |
| **Clinic** | *n* | *n (%)* | *Mean (sd)* | *Mean (sd)* |
| Control | 162 | 99 (61.1) | 11.7 (2.9) | 44.2 (10.3) |
| ASD - ADHD | 139 | 105 (75.5) | 12.1 (3.2) | 57.6 (6.0) |
| ASD + ADHD | 122 | 100 (82.0) | 11.4 (2.9) | 70.1 (6.3) |
| Inattentive | 14 | 11 (78.6) | 9.8 (2.4) | 65.1 (7.0) |
| Hyperactive | 56 | 41 (73.2) | 11.9 (2.8) | 67.6 (5.2) |
| Combined | 52 | 48 (92.3) | 11.3 (3.1) | 74.1 (4.8) |
| **Community** |  |  |  |  |
| Control | 12,104 | 5,829 (48.2) | 11.0 (2.8) | 48.6 (8.9) |
| ASD - ADHD | 91 | 81 (89.0) | 11.3 (2.5) | 55.4 (6.8) |
| ASD + ADHD | 53 | 45 (84.9) | 10.7 (2.1) | 68.1 (5.9) |
| Inattentive | 14 | 12 (85.7) | 10.8 (1.7) | 64.5 (5.3) |
| Hyperactive | 17 | 13 (76.5) | 10.1 (2.2) | 64.9 (4.9) |
| Combined | 22 | 22 (90.9) | 11.1 (2.2) | 72.9 (3.1) |

**Supplemental Table 5: Tests of Group interaction effects**

|  | Community | Clinic |
| --- | --- | --- |
|  | Group x Age | |
| SSRT | F(3,2949) =0.83, p=.5 | F(2,755)=0.09, p>.9 |
| RTV | F(3,3277)=1.00, p=.4 | F(2,838)=1.20, p=.3 |
| RT | F(3,3276)=0.98, p=.4 | F(2,840)=0.42, p=.7 |
|  | Group x Sex | |
| SSRT | F(3,2949) = 2.05, p=.1 | F(2,754)=0.76, p=.5 |
| RTV | F(3,3276) = 1.84, p=.1 | F(2,837)=0.24, p=.8 |
| RT | F(3,3276) = 2.08, p=.1 | F(2,839)=0.24, p=.8 |
|  | Group x SWAN | |
| SSRT | F(3,2945)=1.21, p=.3 | F(2,747)=2.93, p=.054 |
| RTV | F(3,3275)=0.75, p=.5 | F(2,829)=1.57, p=.2 |

**Supplemental Table 6:** Effect of ADHD High Trait on SSRT and RTV in Community Sample.

|  | **SSRT** | | **RTV** | |
| --- | --- | --- | --- | --- |
|  | *% difference*  *(95% CI)* | *p* | *% difference*  *(95% CI)* | *p* |
| High Trait vs Controls | 13.9 (7.4,20.8) | <.0001 | 7.1 (2.9,11.4) | .0007 |
| ASD vs ADHD High Trait | -2.4 (-11.7,7.9) | .6 | 4.0 (-2.6,11.1) | .2 |
| ADHD vs ADHD High Trait | -3.3 (-9.9,3.8) | .4 | 0.1 (-4.4,4.7) | >.9 |

^1^ SSRT models control for Disorder (ADHD, ASD, High Trait and Controls), age, age^2^, gender and stimulant medication; RTV models control for Disorder (ADHD, ASD, High Trait and Controls), age, age^2^, gender and its interaction with age and age^2^

**Supplemental Table 7:** Correlation (95%CI) of SSRT and RTV, full sample and by age.

|  | Community | Clinic |
| --- | --- | --- |
|  | Full sample | |
| Control | 0.07 (0.06, 0.09) | 0.05 (-0.11, 0.20) |
| ASD | 0.28 (0.11, 0.44) | 0.17 (0.04, 0.29) |
| ADHD | 0.20 (0.13, 0.27) | 0.05 (-0.06, 0.15) |
| High ADHD Trait | 0.23 (0.10, 0.35) |  |
|  | Age < 11 years | |
| Control | 0.17 (0.15, 0.20) | 0.13 (-0.11, 0.36) |
| ASD | 0.42 (0.18, 0.61) | 0.35 (0.16, 0.52) |
| ADHD | 0.28 (0.18, 0.38) | 0.04 (-0.09, 0.17) |
| High ADHD Trait | 0.38 (0.23, 0.52) |  |
|  | Age ≥ 11 years | |
| Control | -0.04 (-0.07, -0.02) | -0.06 (-0.26, 0.15) |
| ASD | 0.18 (-0.07, 0.40) | 0.02 (-0.15, 0.19) |
| ADHD | 0.14 (0.04, 0.23) | 0.06 (-0.11, 0.23) |
| High ADHD Trait | 0.09 (-0.11, 0.27) |  |
